# A Dual Reward-Place Association Task to Study the Preferential Retention of Relevant Memories in Rats

**DOI:** 10.3389/fnbeh.2020.00069

**Published:** 2020-05-14

**Authors:** Frédéric Michon, Jyh-Jang Sun, Chae Young Kim, Fabian Kloosterman

**Affiliations:** ^1^Neuroelectronics Research Flanders (NERF), Leuven, Belgium; ^2^Brain and Cognition, KU Leuven, Leuven, Belgium; ^3^VIB-KU Leuven Center for Brain & Disease Research, Leuven, Belgium; ^4^Interuniversity Microelectronics Centre (IMEC), Leuven, Belgium; ^5^Department of Biomedical Sciences, Seoul National University College of Medicine, Seoul, South Korea

**Keywords:** behavior, memory, rats, reward, selective retention

## Abstract

Memories of past events and common knowledge are critical to flexibly adjust one’s future behavior based on prior experiences. The formation and the transformation of these memories into a long-lasting form are supported by a dialogue between populations of neurons in the cortex and the hippocampus. Not all experiences are remembered equally well or equally long. It has been demonstrated experimentally in humans that memory strength positively relates to the behavioral relevance of the associated experience. Behavioral paradigms that test the selective retention of memory in rodents would enable further investigation of the neuronal mechanisms at play. We developed a novel paradigm to follow the repeated acquisition and retrieval of two contextually distinct, yet concurrently learned, food-place associations in rats. We demonstrated the use of this paradigm by varying the amount of reward associated with the two locations. After delays of 2 h or 20 h, rats showed better memory performance for experience associated with large amount of reward. This effect depends on the level of spatial integration required to retrieve the associated location. Thus, this paradigm is suited to study the preferential retention of relevant experiences in rats.

## Introduction

Memory is the ability of the brain to encode and store information for later use. The ability to remember past events and facts, is critically dependent on the medial temporal lobe and its connections to the cortex (Squire et al., 2004). Following initial formation (encoding), a memory trace undergoes active post-processing that stabilizes the trace and integrates it into the brain’s existing knowledge base (consolidation). Both encoding and consolidation are supported by the coordinated activity of neuronal ensembles in the hippocampus and cortical areas (Battaglia et al., 2011). Memory consolidation predominantly occurs during sleep. It engages a bidirectional cortico-hippocampal dialogue characterized by the occurrence of cortical slow wave oscillations, spindles and hippocampal sharp wave ripples (SWRs) (Todorova and Zugaro, 2020).

However, not all experiences are remembered equally well or equally long. A growing body of literature in humans has shown that behaviorally relevant aspects of experience, such as emotional content or expected outcome during learning (Payne et al., 2008; Igloi et al., 2015; Wamsley et al., 2016; Studte et al., 2017), enhance the retention of the associated memory (Stickgold and Walker, 2013). Relevant material is preferentially remembered even in comparison to neutral material occurring concomitantly or close in time. Moreover, the enhanced retention of such experiences correlates with the increased hippocampal activity during learning, as well as post learning (Rauchs et al., 2011; Gruber et al., 2016) and with the increased occurrence of slow wave sleep and spindles in the cortex (Stickgold and Walker, 2013; Igloi et al., 2015; Gruber et al., 2016; Studte et al., 2017). Thus, the enhanced retention of relevant experiences is an active selection process relying on the modulation of the neuronal activity supporting both encoding and consolidation.

Rodents are commonly used as animal models to study cognitive functions and in particular the neurobiology of learning and memory. They present a combination of several advantages. First, rodents require relatively few resources to maintain and can be trained in various behavioral assays (Tolman, 1948; Hodges, 1996; Rosenfeld and Ferguson, 2014; Wood et al., 2018). Second, they share anatomical and functional similarities with humans, especially for key brain regions supporting learning and memory such as the medial temporal lobe (Eichenbaum et al., 2007). Finally, well established tools and techniques exist to monitor neuronal activity, via electrophysiological recordings or optical imaging (Kloosterman et al., 2009; Buzsáki et al., 2015; Ziv and Ghosh, 2015; Weisenburger and Vaziri, 2018), and to manipulate activity in order to determine causal links with the animals’ behavior (Girardeau et al., 2009; Buzsáki et al., 2015; Latchoumane et al., 2017). Aspects of experience, such as rewarded outcomes, have also been shown to affect memory retention in rodents (Salvetti et al., 2014). However, to our knowledge, no study has reported the selective retention of memory for experiences occurring concomitantly.

We developed a novel paradigm to study the selective retention of memories in rodents in which the retention of two, concomitantly acquired food-place associations is assessed every day. This behavioral paradigm was successfully used to confirm the improved memory retention of experiences associated with large reward size, and further, to demonstrate the causal role of post-learning hippocampal replay in the reward-related enhancement of memory consolidation (Michon et al., 2019).

## Materials and Equipment

### Animals

A total of 23 male Long Evans rats (Janvier, France) were food deprived to 85–90% of their free-feeding weight. Of these, 13 animals received an implant for electrical recording (as part of another study), and 10 rats did not undergo surgical procedures and were only tested behaviorally. Upon arrival, the 10 weeks old animals were housed in the animal facility in pairs to acclimate for 1 week. In the following week, the animals were placed in individual housing and on food restriction. In addition, the rats were gently handled for at least 5 min every day to reduce stress and habituate to the experimenters. The animals were kept on a normal light cycle throughout the entire time of the experiment. All experiments were carried out in accordance with protocols approved by KU Leuven Animal Ethics Committee (P119/2015) in accordance with the European Council Directive, 2010/63/EU.

### Apparatus

**FIGURE 1:**
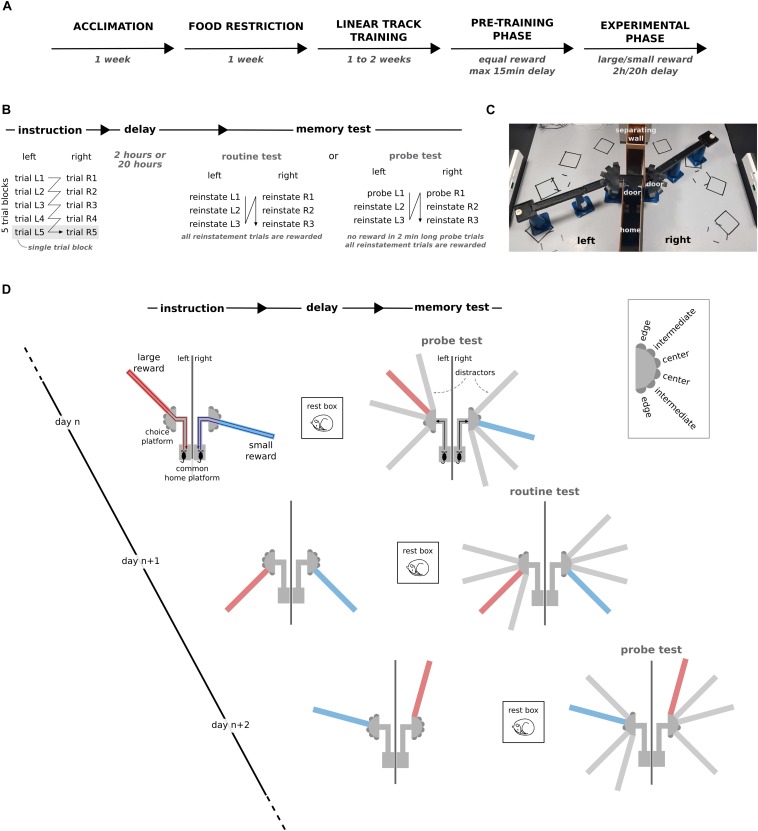
Dual reward-place association task. **(A)** Schematic representation of the experimental timeline. **(B)** Schematic breakdown of the different phases and trials for one training session. **(C)** Photograph of the apparatus during the instruction phase of an example session. **(D)** The behavioral task is composed of three phases: instruction, delay, and memory probe test. During instruction, rats learn to associate a small reward (blue) or a large reward (red) with a specific target arm in the left and right environment. During the memory test after the delay, the preference for the target arm in presence of three distractor arms is assessed as a measure of memory. Inset: labels for target arms based on their location relative to the separating wall. The first trial after the delay phase was either a reinstatement trial or a memory probe trial, and this was alternated from session to session. Across sessions, the location of the rewarded target arms, the configuration of the distractors and the small/large reward assignment to the left/right environment were varied pseudo-randomly.

The behavioral testing apparatus was located in one of two 4 m by 4 m rooms with black walls and distinctive extramaze distal cues (various black and white geometrical shapes printed on white paper) were attached to each of the walls. The apparatus was elevated 40 cm off the ground and consisted of a home platform that gave access to the left and right side of the room via a short 30 cm track (Figures 1C,D). The left and right environments were separated by 120 cm high dividers. In each environment, a choice platform gave access to a maximum of 6 radially emanating 90 cm long arms separated by 30°. Access from the home platform to the two environments was controlled by a door that was manually positioned by the experimenter. Food dispensers were positioned at the end of every arm. To prevent the animals from using olfactory cues to navigate the maze, the food dispensers contained an inaccessible compartment that was filled with the same reward as used for the training that could only be smelled by the animals. Moreover, the maze floor was covered with rubber sheets that were cleaned with water and pseudo-randomly swapped throughout the training sessions.

## Methods

### Behavioral Task

The goal of the task was for the rat to associate one of the 6 arms in each environment with a reward. In each daily session, one environment was associated with a large reward (9 pellets) and the other with a small reward (1 pellet). After the instruction phase, during which the animal could explore the rewarded target arms across five instruction trials per environment, and after a subsequent 2 h or 20 h delay, the rats were tested for their memory of the reward-place association in the presence of three distractor arms (Figure 1D). Across sessions, the location of the target and distractor arms and the assignment of large/small reward size were varied pseudo-randomly (Figure 1D).

### Behavioral Procedure

Prior to experimental sessions, the animals were trained to run back and forth on an elevated linear track (40 cm high and 90 cm long) to obtain food rewards (3 pellets) until the animals executed at least 20 laps within 10 min for three sessions in a row (Figure 1A). During this phase the animals were also habituated to being constrained every time they reached one end of the maze by a door manually controlled by the experimenter.

Next, the rats were familiarized with the experimental procedure and the maze environment of the dual reward-place association task (pre-training phase). During the instruction phase, only the two target arms were physically present in the two environments. The instruction phase consisted of five blocks of alternating trials to the right and left environment. Each trial began with the animal constrained to the home platform. It was then given access to only one of the two environments. The following trial started after the rat had consumed the reward at the end of the target arm and returned to the home platform. The presentation order of the environments within the trial blocks was constant within a session and randomized across sessions. After the instruction phase and before the memory test phase, the rat was removed from the maze apparatus for a short delay and kept in his home cage (as long as needed to add distractor arms to the maze, but at most 15 min). After the delay, rats were subjected to three reinstatement trials separately for the two environments in the presence of the distractor arms (routine test). In each reinstatement trial, rats were rewarded for visiting the target arm with 3 pellets. The aim of the reinstatement trial was for the animal to learn to seek for a reward at the end of the target arm and to ignore the distractor arms. Each reinstatement trial lasted until the animal consumed the reward at the end of the target arm (Figures 1B,D).

The pre-training phase ended when the rats first visited the target arm during the first reinstatement trial in both environments for 3 days in a row. During the experimental phase, the rules of the dual reward-place association task and topography of the maze were kept the same, but different reward sizes (1 and 9 pellets) and longer delays (2 h or 20 h) were introduced. During the delay phase, the rat was either returned to its home cage or placed in a 40 cm × 40 cm rest box with 60 cm high walls that was located inside the behavioral room. In one out of every two sessions, the routine memory test was replaced by a probe memory test. During the probe test, the first reinstatement trial was replaced by a 2-min-long unrewarded memory probe trial separately for the large and small reward environments. After pauses in training (e.g., during weekends), the subsequent experimental session was always preceded by a pre-training session. This procedure was followed to make sure that the rats retained their motivation to search for reward at the target arm in the memory probe trials (Figures 1B,D).

The animals underwent an average of 13 pre-training sessions during the pre-training phase (inter-quartile range: 9–18). On average, the experimental phase lasted for 51 sessions (inter-quartile range: 37–67) including preceding pre-training sessions. The 20 h delay was systematically introduced for animals first tested after 2 h delay (on average for 31 sessions including pre-training sessions, inter-quartile range: 23–33).

### Data Analysis

Data analysis was performed using Python (Millman and Aivazis, 2011) extended with custom toolboxes.

#### Behavior

In the instruction trial, the average running speed to and from the reward platform was quantified only for implanted animals, based on video tracking of the head-mounted LEDs. Average speed was computed over the journey that started when the animal left the home platform and ended when the animal reached the reward platform at the end of a target arm (and vice-versa for the homebound journey).

In the memory probe trial, the number and pattern of visits to the target and distractor arms were quantified as measures of performance in the reward-place association task. A visit to an arm was only counted if the animal reached the reward platform at the end of an arm. We defined the following quantities and (conditional) probabilities to characterize the reward-seeking behavior in the probe trial:

$$N_{v\,i\,s\,i\,t\,s}$$

The total number of arm visits in the 2-min memory probe trial.

$$p\left(visit_{1}=T\right)$$

The across session mean probability that the first visit is on target.

p⁢(T)

The across session mean probability that a visit is made to the target, computed by averaging the equivalent per session *p*(*T*). This probability is further split in the conditional probabilities *p*(*T*|*D*) and *p*(*T*|*T*) that measure the mean probability of visiting the target arm given that the immediately preceding arm visit was also on target [*p*(*T*|*T*)] or was to a distractor [*p*(*T*|*D*)].

$$p\,\left(D_{k}|D_{k}\right)$$

The across session mean probability that a repeat visit is made to any one of the three distractor arms, computed by averaging the equivalent per session.

#### Statistics

To test a difference in two sample means, we used either the Wilcoxon signed rank test (for paired samples) or the Welch’s *t*-test. To test for a difference in two sample proportions, we used either the McNemar test (for paired samples) or the two-proportion *z*-test.

To analyze the dependence of behavioral metrics on predictor variables, we fitted Bayesian generalized linear models (GLMs) using the PyMC3 package for Python (Salvatier et al., 2016). We applied a Poisson regression model (with log link function) for the number of arm visits, a logistic regression model (with logit link function) for *p*(*visit*_1_ = *T*) and an ordinary linear regression model for *p*(*T*).

Model fitting and inference was performed using Markov chain Monte Carlo (MCMC) sampling methods in PyMC3 (specifically, the No-U-Turn Sampler). Broad normal distributions were used as priors on the parameters.

## Results

### Fast Learning of Reward-Place Associations

**FIGURE 2:**
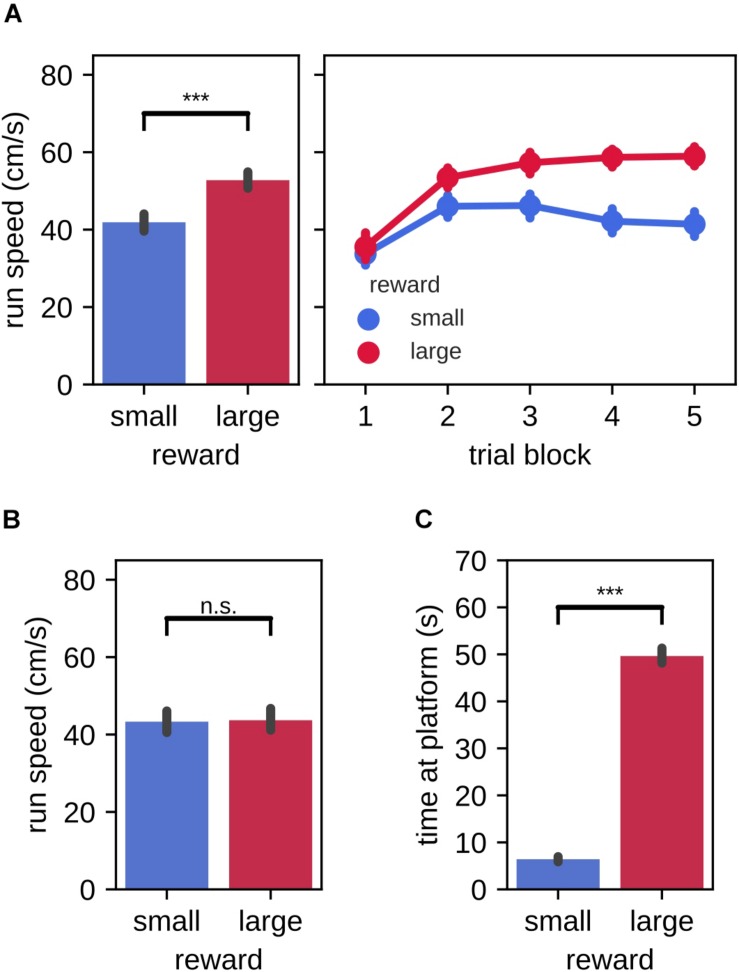
Run speed and time spent at the reward platform during instruction trials. **(A)** Run speed from home to the reward platform. Left: trial-averaged run speed for small and large reward targets. Right: per trial averaged run speed for small and large reward targets. **(B)** Trial-averaged run speed from the reward platform to home for small and large reward targets. **(C)** Trial-averaged time spent at the small and large reward platforms. Error bars represent the 99% confidence interval, ***p < 0.001; n.s., non-significant.

In the instruction phase of the task, we first asked whether the behavior of the rats differed between large and small reward instruction trials, as evidence of fast acquisition of the association between reward magnitude and targets in left/right environment. Indeed, the average running speed toward the reward platform was significantly higher in instruction trials for the large reward amount as compared to the small reward amount [Figure 2A, left; mean (99% CI), large: 52.78 cm/s (50.62,54.91), small: 41.91 cm/s (39.67,44.11); Wilcoxon signed-rank test: *Z* = 707.00, *p* = 2.3×10^−19^]. When analyzed separately for each of the 5 trial blocks, we observed that running speed was low in the first trial block and increased in the second trial block for both large and small reward conditions (Figure 2A, right). Subsequently, running speed remained elevated for the large reward trials and decreased for the small reward trials. No difference between reward conditions was observed for the running speed from the reward platform back home [Figure 2B, mean (99% CI), large: 43.70 cm/s (40.84,46.68), small: 43.33 cm/s (40.64,46.09); Wilcoxon signed-rank test: *Z* = 5056.00, *p* = 0.74]. Rats also spent more time consuming the reward on the platform at the end of the target arm that was associated with large reward as compared to small reward [Figure 2C, mean (99% CI), large: 49.64 s (48.05,51.35), small: 6.41 s (5.88,6.97)].

### Stronger Behavioral Bias Toward Target Arms Associated With Large Reward Amount After 2 h Delay

Overall, 23 rats performed a total of 151 sessions of probe test following 2 h of delay. During the probe trials, rats made a median of 8 arm visits (inter-quartile range: 6–10, 151 sessions in 21 animals). As we reported previously (Michon et al., 2019), rats were more likely than chance to visit the target arm on their first journey and throughout the 2 min of the probe trials for both reward environments. The performance for the large reward condition, however, was higher than for the small reward condition.

**FIGURE 3:**
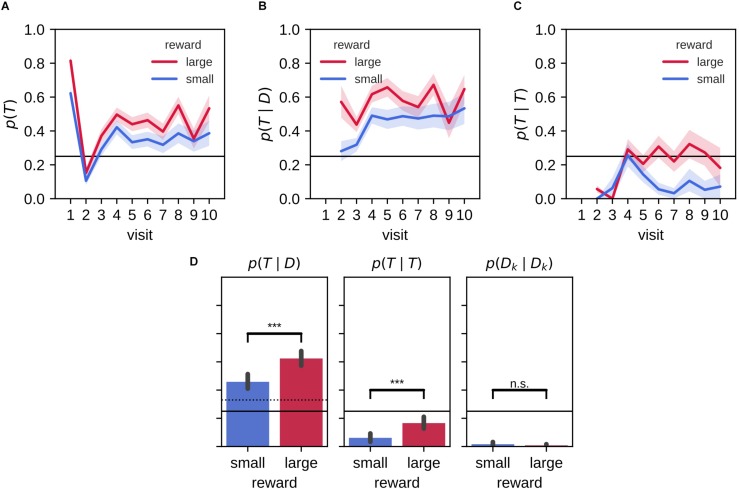
Stronger bias for large reward-place associations after 2 h delay. **(A)** Probability of visiting a target arm is higher across visits in the large reward environment compared to the small reward environment. On the first visit, animals often go straight to the target arm followed by a visit to a distractor arm on the second visit. On the remaining visits, the probability to visit the target arm remains constant. **(B)** The conditional probability of visiting the target arm given that the animal previously visited a distractor arm across visit is higher for large reward than small reward. **(C)** The probability of a repeat visit to the target arm is low for initial visits but increases afterward. For the large reward condition, this increase persists while for the small reward condition the probability of making a repeat visit declines. **(D)** The associated conditional probabilities of visiting the target arm given that the rat previously visited a distractor arm (left) or the target arm (middle; i.e., repeat visit to the target arm) and the probability for a repeat visit to the same distractor arm (right). Error bars represent the 99% confidence interval; solid line: 0.25 chance level, dashed line: $0.\bar{3}$ chance level, ***p < 0.001; n.s., non-significant.

We looked in more detail at the behavior during the memory probe trial by separately analyzing the target arm preference for the first arm visit and subsequent visits (Figure 3A). On their first journey, rats were more likely than chance level (*p* = 0.25) to visit the target arm [*p*(*visit*_1_ = *T*); Figure 3A]. The preference for the target on the first visit was stronger for the large reward environment than the small reward environment. On the second visit, rats had a higher tendency to explore non-target arms, before a clear preference to revisit the target arm established in the remainder of the 2-min probe trial.

We computed the conditional probability *p*(*visit*_*k*_ = *T*|*visit*_*k*−1_ = *D*) = *p*(*T*|*D*) where *T* indicates the visit to a target arm and *D* to a distractor arm (Figure 3B). In both large and small reward conditions, the revisit probability *p*(*T*|*D*) is significantly higher than naive chance level (0.25) and the more conservative chance level of $0.\bar{3}$ that assumes rats never immediately return to the exact same arm they just visited [Figure 3D, left; mean (99% CI), large: 0.62 (0.57,0.67), small: 0.46 (0.41,0.51)]. The target revisit probability is significantly higher for the large reward environment than the small reward environment [mean large-small difference (99% CI): 0.17 (0.10,0.23); Wilcoxon signed-rank test: *Z* = 1913.50, *p* = 2.5×10^−9^].

Rats have a natural tendency to alternate maze arms and to avoid visiting the same arm twice in succession. Accordingly, rats made virtually no repeat visits to the same distractor arm [Figure 3D, right; repeat probability *p*(*D*_*k*_|*D*_*k*_); mean (99% CI), large: 0.01 (0.00,0.02), small: 0.02 (0.00,0.03)]. However, rats did show an increased tendency to immediately return to the target arm without visiting any other arm in between, expressed as the conditional probability *p*(*T*|*T*) (Figure 3C). Interestingly, an increase of the repeat visit probability *p*(*T*|*T*) developed from the fourth visit in both small and large reward conditions, but the increase was only temporary for the small reward condition. On average the probability *p*(*T*|*T*) was lower than chance for both large and small reward conditions [Figure 3D, middle; mean (99% CI), large: 0.17 (0.12,0.21), small: 0.06 (0.03,0.09)], but they were significantly higher than the corresponding probability of repeat visits to distractor arms *p*(*D*_*k*_|*D*_*k*_). Moreover, *p*(*T*|*T*) was significantly higher for the large reward as compared to the small reward condition [Figure 3D, middle; mean large-small difference (99% CI): 0.11 (0.06,0.15); Wilcoxon signed-rank test: *Z* = 426.50, *p* = 5.9×10^−7^].

### Influence of Spatial Configuration

We next tested if other factors also contributed to the behavioral performance. Due to asymmetries in the configuration of target and distractor arms, the spatial configurations experienced varies between sessions. As reported in Michon et al. (2019), the probability of the rats to first visit the target arm was robust to the centrality of the target arm when associated with large reward, but increased from central to edge target arm locations in the small reward condition.

**FIGURE 4:**
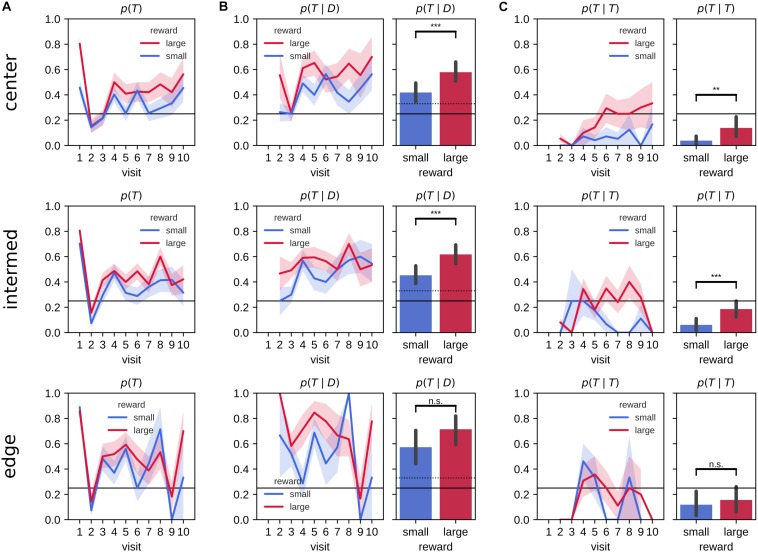
Probabilities of visits to the target arm separated by target arm location. **(A)** Probability of visiting a target arm is higher across visits in the large reward environment compared to the small reward environment. On the first visit, animals often go straight to the target arm followed by a visit to a distractor arm on the second visit. The difference between large and small reward environments is clearly visible for central target arms, but not edge target arms. On visits three and up, the animals developed a preference for revisiting the target arm (over distractor arms), which is stronger in large reward environment when the target is in a central or intermediate but not edge location. **(B)** The conditional probability of visiting the target arm given that the animal previously visited a distractor arm across visit (left) and associated mean probability (right) is larger in the large reward environment compared to the small reward environment for center and intermediate target arm locations. **(C)** The conditional probability of a repeat visit across visits (left) and associated mean (right). Rats are more likely to repeat visits to the target arm in the large reward environment compared to small reward environment only for center and intermediate, but not edge, target arm locations. Error bars represent the 99% confidence interval; solid line: 0.25 chance level, dashed line: $0.\bar{3}$ chance level, **p < 0.005; ***p < 0.001; n.s., non-significant.

We next looked at the effect of the target arm locations on the evolution of the visit preference (Figure 4A). The difference in the probability of first visiting the target arm *p*(*visit*_1_ = *T*) between large and small reward conditions was largest for central arms, whereas for edge arm locations performance did not differ between reward conditions. For all target arm locations, rats had a higher tendency to explore non-target arms on their second visits and to revisit more the target arm in the remainder of the 2-min probe trial. However, the animals developed a preference for revisiting the target arms associated with the large reward amount for the center and intermediate, but not the edge locations.

We quantified the conditional probabilities, revisit probability *p*(*T*|*D*) (Figure 4B) and repeat visit probability *p*(*T*|*T*) (Figure 4C) separately for central, intermediate and edge target arm locations. The mean difference in revisit probability *p*(*T*|*D*) was similar for all locations [mean difference (99% CI) for large-small reward at center, intermediate and edge arm locations, center: 0.16 (0.05,0.27), intermediate: 0.16 (0.06,0.27) and edge: 0.14 (−0.04,0.32)] but was only statistically significant for center and intermediate locations, due to a higher variability for the edge locations [two sample means Welch’s *t*-test for large-small reward differences, with Bonferroni corrected *p*-value for three hypotheses, center: *t*(115) = −3.81, *p* = 0.00024, intermediate: *t*(130) = −4.09, *p* = 7.7×10^−5^, edge: *t*(54) = −2.02, *p* = 1]. During the probe trials, rats were more likely to repeat visit to target arms *p*(*T*|*T*) associated to large reward amount compared with small reward for the center and intermediate locations, but not the edge locations [mean difference (99% CI) for large-small reward at center, intermediate and edge arm locations, center: 0.10 (0.03,0.18), intermediate: 0.12 (0.05,0.20) and edge: 0.04 (−0.11,0.19); two sample means Welch’s *t*-test for large-small reward differences, with Bonferroni corrected *p*-value for three hypotheses, center: *t*(110) = −3.22, *p* = 0.002, intermediate: *t(*129) = −4.10, *p* = 7.4×10^−5^, edge: *t*(54) = −0.65, *p* = 1].

### Influence of Presentation Order

**FIGURE 5:**
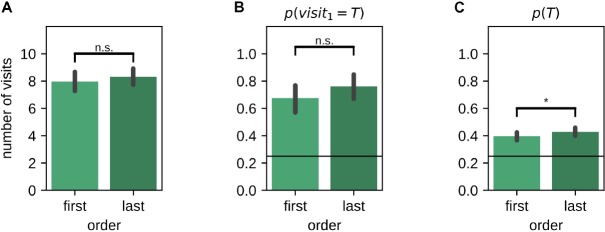
Effect of the order in which left/right environments were presented and tested. **(A)** Number of arm visits in 2-min memory probe trial split by presentation order. **(B)** Probability of visiting the target arm on the first visit in the 2-min memory probe trial split by presentation order. **(C)** Average probability of visiting the target arm in the 2-min memory probe trial split by presentation order. Error bars represent the 99% confidence interval; *p < 0.05; n.s., non-significant.

The presentation order of the left and right environments in the instruction trials and memory probe trials was randomized across sessions. While the total number of visits [mean last-first difference (99% CI): 0.34 (−0.22,0.87); Wilcoxon signed-rank test: *Z* = 3285.50, *p* = 0.059] (Figure 5A) and the on-target probability of the first visit *p*(*visit*_1_ = *T*) during the memory probe [mean last-first difference (99% CI): 0.09 (−0.04,0.21); McNemar test, *H*_0_:*p*_*first*_ = *p*_*last*_, χ^2^ = 21.00, *p* = 0.1] (Figure 5B) were not affected by the presentation order, the average target visit probability *p*(*T*) was marginally lower for the environment tested first [mean last-first difference (99% CI): 0.03 (−0.01,0.07); Wilcoxon signed-rank test: *Z* = 3968.50, *p* = 0.033] (Figure 5C).

### Effect of Repeated Experience in the Task

**FIGURE 6:**
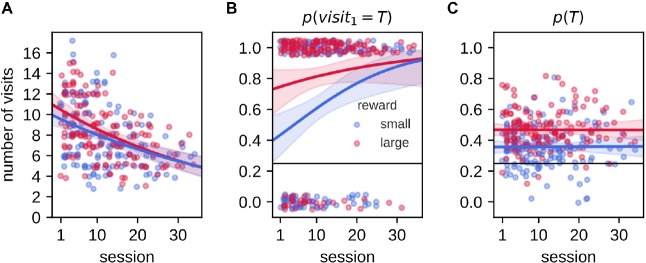
Effect of accumulated experience on behavioral performance. In all panels, individual data points are shown as small dots (with added jitter to reduce overlap), line and shaded region represent the mean and 95% credible region of posterior predictive samples from Bayesian GLM model fit. **(A)** The number of arm visits in 2-min memory probe trial decreases as a function of experimental session for small and large reward environments. Data are fitted with Poisson regression model. **(B)** The probability of visiting the target arm on the first visit in the 2-min memory probe trial increases with experience only for the small reward condition. Data are fitted with logistic regression model. **(C)** The average probability of visiting the target arm *p*(*T*) in the 2-min memory probe trial does not change with experience. Data are fitted with linear regression model.

Rats were pre-trained to be familiar with the task rules and subsequently repeatedly trained/tested with daily varying reward-place associations for several weeks. We asked if the animals’ performance varied over time. With increasing experience in the task, the total number of arm visits during the 2-min memory probe trial decreased for both reward conditions [Figure 6A, Poisson regression model, *slope*_*small*_: −0.019 (−0.027, −0.011), *slope*_*large*_: −0.022 (−0.03, −0.015)]. This indicates that the animals reduced their reward-seeking behavior, possibly because they learned to recognize a memory probe trial that is never rewarded. The probability *p*(*visit*_1_ = *T*) increased significantly across sessions, but only for the small reward condition [Figure 6B, Logistic regression model, *slope*_*small*_: 0.075 (0.027,0.13), *slope*_*large*_: 0.037 (−0.014,0.1)]. In contrast, the decrease in total number of visits did not affect the average probability *p*(*T*) to visit the target arm [Figure 6C, ordinary least squares regression model slope_small : −2.6×10^−5^ (−0.0025,0.0028), *slope*_*large*_: −5×10^−5^ (−0.0028,0.0026)]. Thus, while rats were familiar with the basic task rules prior to the experimental phase, we still observed an increase of performance across sessions, possibly because the introduction of small/large reward size and extended delay.

### Difference in Performance Between Large and Small Reward Conditions Partially Maintained After 20 h Delay

**FIGURE 7:**
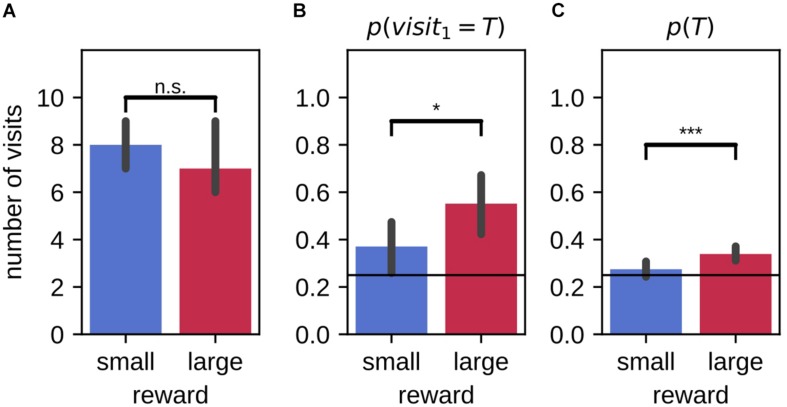
Difference in performance between large and small reward environments are partially maintained after a 20 h delay. **(A)** On average, animals make a similar number of arm visits in the 2-min probe trial in the two environments. **(B)** For all test sessions (probe and routine test) combined, the probability that animals first visit the target arm in the 2-min probe trial is higher in the large reward environment as compared to the small reward environment. Performance for both large and small environments remains above chance. **(C)** The average probability of visiting the target arm in the 2-min probe trial is higher in the large reward condition compared to the small reward condition. Performance for large reward, but not for small reward, is above chance. Error bars represent the 99% confidence interval; solid line: 0.25 chance level, dashed line: $0.\bar{3}$ chance level, *p < 0.05; ***p < 0.001; n.s., non-significant.

Rats were also tested for their memory of the target arm locations after 20 h delay (9 animals, 59 sessions). They made similar number of total visits within the 2-min probe trials in large and small reward environments [Figure 7A, mean large-small difference (99% CI): −0.15 (−0.95,0.61); Wilcoxon signed-rank test: *Z* = 613.50, *p* = 0.82]. Overall, the preference for visiting the target arm (over distractor arms) was stronger in the large reward condition as compared to the small reward condition [Figure 7C; mean large-small difference (99% CI): 0.06 (0.02,0.11); Wilcoxon signed-rank test: *Z* = 314.00, *p* = 0.00023]. The average probability to visit the target arm *p*(*T*) was only higher than chance in the large reward condition (10000 simulations; Monte-Carlo *p*-value, large: p = 0.0001, small: *p* = 0.14). On their first journey, the probability of visiting the target arm *p*(*visit*_1_ = *T*) was higher than chance level for both large and small reward conditions [*p* = 0.25; mean (99% CI), large: 0.53 (0.36,0.69), small: 0.41 (0.25,0.58); binomial test under the null hypothesis of uniform arm visit probability, large: *p* = 6.2×10^−6^, small: *p* = 0.0097]. Despite a tendency for the probability of first visiting the target arm associated to the large reward to be higher than for the small, the difference between the two reward conditions was not significant when including the probe memory test sessions only [mean large-small difference (99% CI): 0.12 (−0.14,0.37); McNemar test, *H*_0_:*p*_*small*_ = *p*_*larg*⁡*e*_, χ^2^ = 14.00, *p* = 0.31]. However, the conditions in which the rats choose the first arm to visit are the same between probe and routine test sessions. When combining all test sessions, the difference between reward conditions was then significant [Figure 7B; probe and routine test, 116 sessions; mean large-small difference (99% CI): 0.18 (0.01,0.35); McNemar test, *H*_0_:*p*_*small*_ = *p*_*larg*⁡*e*_, χ^2^ = 23.00, *p* = 0.014].

## Discussion

Our aim was to develop a behavioral paradigm to study the enhanced memory retention of salient experiences in rodents. Each day, rats were trained to learn a different food-place association on two contextually distinct semi-radial arm mazes. One arm was associated with a large amount of reward, and the other with a small amount of reward. The locations of the arm and the reward associated to the two environments were pseudo-randomly assigned every day. After a delay of 2 h or 20 h the animals were placed again in the two environments separately to be tested on their memory for the previously rewarded locations.

During training, rats showed a rapid increase in average run speed on journeys toward the reward. Moreover, a difference in speed developed throughout the training. The speed on journeys to the large reward remained stable while the speed to the small reward decreased toward the end of the training sessions, suggesting different motivational states consistent with the two different rewarded outcomes. These results indicate that the animals were able to quickly learn the food place associations.

Following a previous study showing that rats remember better locations associated with larger reward amount after 1 and 24 h (Salvetti et al., 2014), the performance of the animals to retrieve the previously rewarded locations was assessed by monitoring the pattern of arm visits followed during the test phase after 2 and 20 h delays. For both delays and both reward conditions, the rats were more likely than chance to first visit the target arm, which indicates that they remembered the food-place associations. The probability of visiting the target arm throughout the 2 min probe test period was also above chance level for all conditions except for the arm associated with small reward after 20 h, possibly reflecting a lesser degree of confidence in their memory for the location of the rewarded arm in this condition. Consistent with the reported negative effect of prolonged delay on memory retention (Murre and Dros, 2015), the animals’ performance was lower after a longer delay of 20 h for both reward conditions. On average, the performance of the animals was also higher for the location of the large reward compared to small the small reward for both delays. These results are consistent with the studies carried out in human showing a selective enhancement of memory for salient experiences, including the expectation of a higher value outcome, after both a short or long retention delay (Igloi et al., 2015; Gruber et al., 2016; Studte et al., 2017). Our results further indicate that, after a 2 h delay, a large rewarding outcome drove the animals away from their natural alternating behavior, as they were more likely to directly repeat a visit to the target arm. Overall, the results validate the use of the dual reward-place associations task to study the mechanisms underlying the selective retention of memory in rodent.

Further analysis revealed that other aspects of the experience in the paradigm influenced the performance of the animals after 2 h of retention delay. The performance varied in function of the location of the baited arm relative to the boundaries of the environments. The more radially distant the arm was from the boundary, the more the performance of the animals decreased. This effect was particularly pronounced in the small reward condition, so that performance for target arms close to the boundary were similar between the two reward conditions and the difference progressively increased for the target arms located centrally. First, these results confirm a modulation of memory retention by the amount of reward as the differences in performance cannot solely be explained by different seeking strategies related to motivational state. Second, it indicates that the reward-related enhancement of memory was interacting with other features of the experience potentially dependent on different memory systems (Ekstrom et al., 2014; Kirch et al., 2015). Rats use different strategies, such as cue-response and allocentric strategies, to navigate an environment. Cue-response and allocentric strategies are known to depend on different brain structures, respectively, the nucleus accumbens and the hippocampus (Packard and McGaugh, 1996). Our previous study (Michon et al., 2019) showing that the performance for the more centrally located arms associated to high reward is sensitive to hippocampal ripples disruption suggest that the ability to consolidate and retrieve the rewarded location is hippocampal-dependent, at least for these reward-location associations. However, this observation does not rule out the fact that the animals may use hippocampal-independent strategies in this paradigm, in particular for other reward-arm configurations. Further experiments, involving the inactivation of the hippocampus or the nucleus-accumbens in rats trained in the dual reward-place association task are necessary to assess the relative contribution of these brain regions in this paradigm.

Despite being highly familiarized with the majority of the task parameters, the performance of the animal improved over time, at the scale of weeks of training (meta learning). The improvement in performance may reflect learning associated with the changes introduced at the start of the experimental procedure, such as the increased retention delays, the different rewarded outcomes or the non-rewarded trials used as probe memory tests. The increase in memory performance was accompanied with a reduction of the seeking behavior during the probe tests, suggesting that the animals had adjusted their behavior to the fact that these trials were unrewarded, which argues in favor of the fact that at least part of the meta learning reflected the learning of the changes in the tasks. The meta learning related to memory performance was mainly observed for the small reward condition, while performance in the large reward conditions were already close to their maximum from the early phase in the training. This suggests that higher value outcome during training accelerates meta-learning.

The dual reward-place association task is suited to study the modulation of memory retention by features of experience as was demonstrated with varying the amount of reward associated to two similar but distinct experiences. However, the current paradigm can be further optimized, in particular, to circumvent two observed limitations. First, the enhancement of memory for highly rewarded experience was dependent on locations presumably requiring the use of an allocentric strategy. The dependence of the reward-enhancement of memory to two third of the arm locations (one third for a maximal effect) reduces the efficacy of the paradigm. Maximizing the number of locations requiring higher level of spatial integration would thus optimize data collection. For example, increasing the total number of possible arm locations would increase the ratio between the number of central and edge arms locations. In addition, the increased density of rewarded location is also expected to more strongly depend on the hippocampus (Clelland et al., 2009). Second, the fact that the animals spent more time consuming the large amount of reward compared to small is a confounding factor in the paradigm. Several approaches could be used to at least mitigate a putative effect of time spent at the reward location on memory retention: use the natural behavior of rats to carry food to consume it in less exposed conditions or use different concentrations of rewarding agents in solution (Whishaw and Dringenberg, 1991; Salvetti et al., 2014). Moreover, most aspects of the task, such as positioning the doors or reward delivery, are currently manually handled by the experimenter. The automation of these aspects would improve reproducibility and decrease variability of the experiment. Finally, the paradigm can be further expanded by modulating other features of experience, such as the hedonic valence associated to the different environments (Perry et al., 2016) or by introducing mild aversive stimuli (Girardeau et al., 2017).

The choice of behavioral assay is critical, not only to ensure a behavioral read out of the cognitive process of interest, but also as it participates in optimizing the amount and reliability of the data collected. Experiments combining neuronal recording and/or manipulations with behavior are critical to understand how brain activity supports cognitive functions, but they require a large investment for each animal. Repetitive and behaviorally constrained paradigms have the potential to increase the outcomes from experiments correlating brain activity with behavior by reducing variability and increasing the number of datasets collected per animal. Moreover, experiment involving manipulations, for example of particular aspects of an experience or directly of brain activity, benefits from the ability to compare the effects with internal controls from the same animals, between or within the same experimental sessions. The dual reward-place association paradigm is suitable for neuronal recordings and manipulations (Michon et al., 2019): the use of radial arms enables to render the behavior of the animals more stereotypical, the training can be repeated over weeks daily changing the place-reward associations and it allows the use of internal controls between and within sessions.

## Data Availability Statement

The datasets generated for this study are available on request to the corresponding author.

## Ethics Statement

The animal study was reviewed and approved by the KU Leuven Animal Ethics Committee (P119/2015).

## Author Contributions

FM and FK conceived and designed the study and wrote the manuscript. FM, CK, and J-JS carried out the experimental work. FM, CK, J-JS, and FK analyzed and interpreted the data. CK and J-JS reviewed the manuscript. FK coordinated and provided funding for the project.

## Conflict of Interest

The authors declare that the research was conducted in the absence of any commercial or financial relationships that could be construed as a potential conflict of interest.
